# A functional outcome prediction model of acute traumatic spinal cord injury based on extreme gradient boost

**DOI:** 10.1186/s13018-022-03343-7

**Published:** 2022-10-12

**Authors:** Zhan Sizheng, Huang Boxuan, Xue Feng, Zhang Dianying

**Affiliations:** 1 Department of Orthopedics, Peking University People’s Hospital, No. 11 Xizhimen South Street, Xicheng District, Beijing, 100044 China; 2 Ministry of Education Key Laboratory of Trauma Treatment and Nerve Regeneration, Peking University People’s Hospital, Beijing, 100044 China; 3 Department of Orthopedics, Peking University Binhai Hospital, Tianjin, 300450 China

**Keywords:** Acute spinal cord injury, Prediction model, Extreme gradient boost, Spinal cord independence measure

## Abstract

**Objective:**

We aimed to construct a nonlinear regression model through Extreme Gradient Boost (XGBoost) to predict functional outcome 1 year after surgical decompression for patients with acute spinal cord injury (SCI) and explored the importance of predictors in predicting the functional outcome.

**Methods:**

We prospectively enrolled 249 patients with acute SCI from 5 primary orthopedic centers from June 1, 2016, to June 1, 2020. We identified a total of 6 predictors with three aspects: (1) clinical characteristics, including age, American Spinal Injury Association (ASIA) Impairment Scale (AIS) at admission, level of injury and baseline ASIA motor score (AMS); (2) MR imaging, mainly including Brain and Spinal Injury Center (BASIC) score; (3) surgical timing, specifically comparing whether surgical decompression was received within 24 h or not. We assessed the SCIM score at 1 year after the operation as the functional outcome index. XGBoost was used to build a nonlinear regression prediction model through the method of boosting integrated learning.

**Results:**

We successfully constructed a nonlinear regression prediction model through XGBoost and verified the credibility. There is no significant difference between actual SCIM and nonlinear prediction model (*t* = 0.86, *P* = 0.394; Mean ± SD: 3.31 ± 2.8). The nonlinear model is superior to the traditional linear model (*t* = 6.57, *P* < 0.001). AMS and age played the most important roles in constructing predictive models. There is an obvious correlation between AIS, AMS and BASIC score.

**Conclusion:**

We verified the feasibility of using XGBoost to construct a nonlinear regression prediction model for the functional outcome of patients with acute SCI, and proved that the predictive performance of the nonlinear model is better than the traditional linear regression prediction model. Age and baseline AMS play the most important role in predicting the functional outcome. We also found a significant correlation between AIS at admission, baseline AMS and BASIC score.

***Trial registration*:**

ClinicalTrials.gov identifier: NCT03103516.

**Supplementary Information:**

The online version contains supplementary material available at 10.1186/s13018-022-03343-7.

## Introduction

Acute traumatic spinal cord injury (SCI) is a severe condition that affects individuals worldwide and is associated with a high rate of disability [1–3]. Acute SCI not only greatly aggravates the economic burden of society, family, and individuals but also exerts great psychological pressure on patients and their families [4, 5]. Prognosticating functional outcome after acute SCI is important to guide management strategies and to give the patients and their families a realistic idea of long-term expectations [6, 7].

In 2012, Wilson and colleagues [6] retrospectively analyzed the clinical and imaging features of patients with acute SCI, used the functional independence measure (FIM) as a functional outcome indicator, and successfully constructed a linear regression model to predict FIM in patients after 1 year. In 2017, Kaminski and colleagues [7] prospectively analyzed the acute phase clinical characteristics of patients with acute SCI, used the Spinal Cord Independence Measure (SCIM) as a functional prognostic indicator, and constructed a linear regression model to predict SCIM in patients after 1 year. However, the prognostic relationship between functional outcome and different indicators is often not a simple linear correlation. At the same time, the in-depth research on SCI treatments, such as early surgery [8–12], has indicated that these measures have a significant impact on the prognosis of patients with acute SCI.

Extreme Gradient Boost (XGBoost) is an open source machine learning project developed by Chen Tianqi et al. [13] in 2016, and it exhibited the most advanced performance in the Kaggle machine learning competition. XGBoost was developed on the basis of Gradient Boosting Decision Tree (GBDT) and is a type of boosting ensemble learning. Ensemble learning refers to the construction of multiple classifiers, such as Classification and Regression Tree (CART), to predict the dataset and then use a certain strategy to integrate results of the multiple classifiers as the final prediction result. As a common method of boosting ensemble learning, every calculation of GBDT is to reduce the last residual and then establishes a new model in the direction of residual reduction (negative gradient). XGBoost is faster and more efficient than GBDT, so it is called X (Extreme) GBoost. Ensemble learning has been widely used in many fields, such as healthcare industry, commerce and environmental protection [14–16].

Here, through a designed prospective cohort study, we included three aspects of potential predictors (clinical features, MR imaging and surgical timing), chose SCIM as the functional outcome indicator, and aimed to construct a nonlinear regression model through XGBoost to predict patient functional outcome 1 year after surgical decompression.

## Methods

### Study cohort

We conducted a prospective, multicenter nonrandomized controlled trial involving five hospitals in Beijing: (1) Peking University People’s Hospital, (2) Peking University Third Hospital, (3) Beijing Friendship Hospital Affiliated to Capital Medical University, (4) Chaoyang Hospital Affiliated to Capital Medical University, and (5) Chinese People’s Liberation Army (PLA) General Hospital. All the hospitals recorded patient information in a database specifically created for SCI cases. Prior to the start of the study, the protocol involving all five hospitals was approved by the ethics committee. We do not routinely use methylprednisolone therapy in our patients due to the uncertainty of methylprednisolone therapy and the high risk of complications [17]. The study was approved by the ethics committee of Peking University People’s Hospital, approval number: 2016PHB136-01.

Inclusion criteria

1. Age: 16–85 years old, irrespective of sex;
2. Final diagnosis by spine magnetic resonance (MR) imaging;
3. Cervical and thoracic fracture dislocation or without fracture dislocation but combined with spinal cord injury;
4. No other injury involving life, injury severity score < 16 [18];
5. Receiving surgical decompression

Exclusion criteria

1. History of mental illness and metal allergy;
2. Long-term alcohol abuse and drug abuse;
3. Did not agree to participate in this trial/the legal representative of the patient refuses to sign informed consent;
4. Refusal to examine and treatment options

A total of 249 patients met all inclusion criteria and were included in the study from June 1, 2016, to June 1, 2020. At the same time, we retrospectively included patients with acute SCI at Tianjin Binhai Hospital from June 1, 2016, to June 1, 2020, as the validation sample set. The inclusion and exclusion criteria and data collection were consistent with the prospective study.

### Predictor variables

The determination of our predictor variables was based on three main principles: (1) the literature proves that the selected variables are related to the patient's functional outcome; (2) the selected variables are easy to obtain in clinical work; (3) the selected variables have good reliability among doctors. Based on these three principles, we identified a total of 6 predictors: (1) clinical characteristics, including age, American Spinal Injury Association (ASIA) Impairment Scale (AIS) at admission, level of injury and baseline ASIA motor score (AMS); (2) MR imaging, mainly including Brain and Spinal Injury Center (BASIC) score; (3) surgical timing, specifically comparing whether surgical decompression was received within 24 h or not (Table 1A). All six predictors have demonstrated prognostic significance in relation to long-term functional outcome after SCI [6–12, 19–21]. A professional orthopedic surgeon conducted physical examinations to identify the patients’ neurologic level of injury, AMS and AIS at admission. MR imaging was the earliest recorded MR result for patients. The MR imaging examinations were performed with a 1.5-Tesla MR scanner (Signa CV/I, GE Healthcare, Milwaukee, WI). We assessed sagittal T2 FSE, sagittal T1, and axial T2 FSE sequences to calculate the BASIC score. Two authors individually and independently assessed the imaging data twice to eliminate intra- and inter-observer bias. The timing of the operation was to truthfully record the time between injury and the operation.

**Table 1:** Predictor and outcome variables

Variables	Description
A. Predictor variabels	
Age	Continuous;
Level of injury	Cervical; Thoracic
AIS at admission	Grade A = 1: no motor or sensory function is preserved in the sacral segments Grade B = 2: sensory but no motor function is preserved below the neurological level and includes the sacral segments Grade C = 3: motor function is preserved below the neurological level, and more than half of key muscles below this level have a muscle grade less than 3 Grade D = 4: motor function is preserved below the neurological level, and more than half of key muscles below this level have a muscle grade of 3 or more
Baseline AMS	Continuous; Rang: 0–100
BASIC score	0 = normal; 1 = GM only; 2 = some WM; 3 = all WM in plane; 4 = with hemorrhage
Time to operation	Eraly surgery (= < 24 h); Delayed surgery (> 24 h)
B. Outcome variables	
SCIM III	
Self care	1. Feeding/3 2. Bathing A. Upper body/3 B. Lower body/3 3. Dressing A. Upper body/4 B. Lower body/4 4. Grooming/3
Respiration and sphincter	5. Respiration/10 6. Sphincter management—bladder/15 7. Sphincter management—bowel/10 8. Use of toilet/5
Mobility (room and toilet)	9. Mobility in bed and action to prevent pressure sores/6 10. Transfers: bed-wheelchair/2 11. Transfers: wheelchair-toilet-tub /2
Mobility (indoors and outdoors)	12. Mobility indoors/8 13. Mobility for moderate distances (10–100 m)/8 14. Mobility outdoors (more than 100 m) /8 15. Stair management/3 16. Transfers: wheelchair-car/2 17. Transfers: ground-wheelchair/1

*GM* grey matter, *WM* white matter

### Outcome and follow-up

We assessed the SCIM score at 1 year after the operation as the functional outcome index. The SCIM score is composed of 19 items, with three main domains (Table 1B): self-care (six items, scores range from 0 to 20); respiration and sphincter management (four items, scores range from 0–40); and mobility (nine items, scores range from 0 to 40). The SCIM score was first proposed by Cate et al. [22] and has now been revised in a third edition [23]. An international multicenter study found that SCIM has good reliability, validity and practicability in people with SCI [24] and is superior to FIM [25].

### Statistical analysis

XGBoost builds a nonlinear regression prediction model through the method of boosting integrated learning. Compared with other boosting ensemble learning, XGBoost can be used to construct predictive models more efficiently and accurately by performing second-order Taylor expansion, regularization term, and optimizing greedy algorithms on the objective function. We implemented XGBoost through Python 3.9. Since our sample data are relatively small, we choose n_estimators = 10,000 (CART) in the XGBoost prediction model. The linear regression prediction model was built by IBM SPSS Statistics for Windows, version 26.0 (IBMCorp., Armonk, N.Y., USA). Mean Square Error (MSE), Mean Absolute Error (MAE) and Mean Absolute Percentage Error (MAPE) were used for the evaluation of predictive models.

$${\text{MSE}} = \frac{1}{n}\mathop \sum \limits_{1}^{n} \left( {\widehat{yi} - yi} \right)^{2}$$

$${\text{MAE}} = \frac{1}{n}\mathop \sum \limits_{1}^{n} \left| {\widehat{yi} - yi} \right|$$

$${\text{MAPE}} = \frac{1}{n}\mathop \sum \limits_{1}^{n} \left| {\frac{{\widehat{yi} - yi}}{yi}} \right|$$

${\text{yi}}$: the real value; $\widehat{yi}$: the predicted value.

## Results

### Study population

**Fig. 1:**
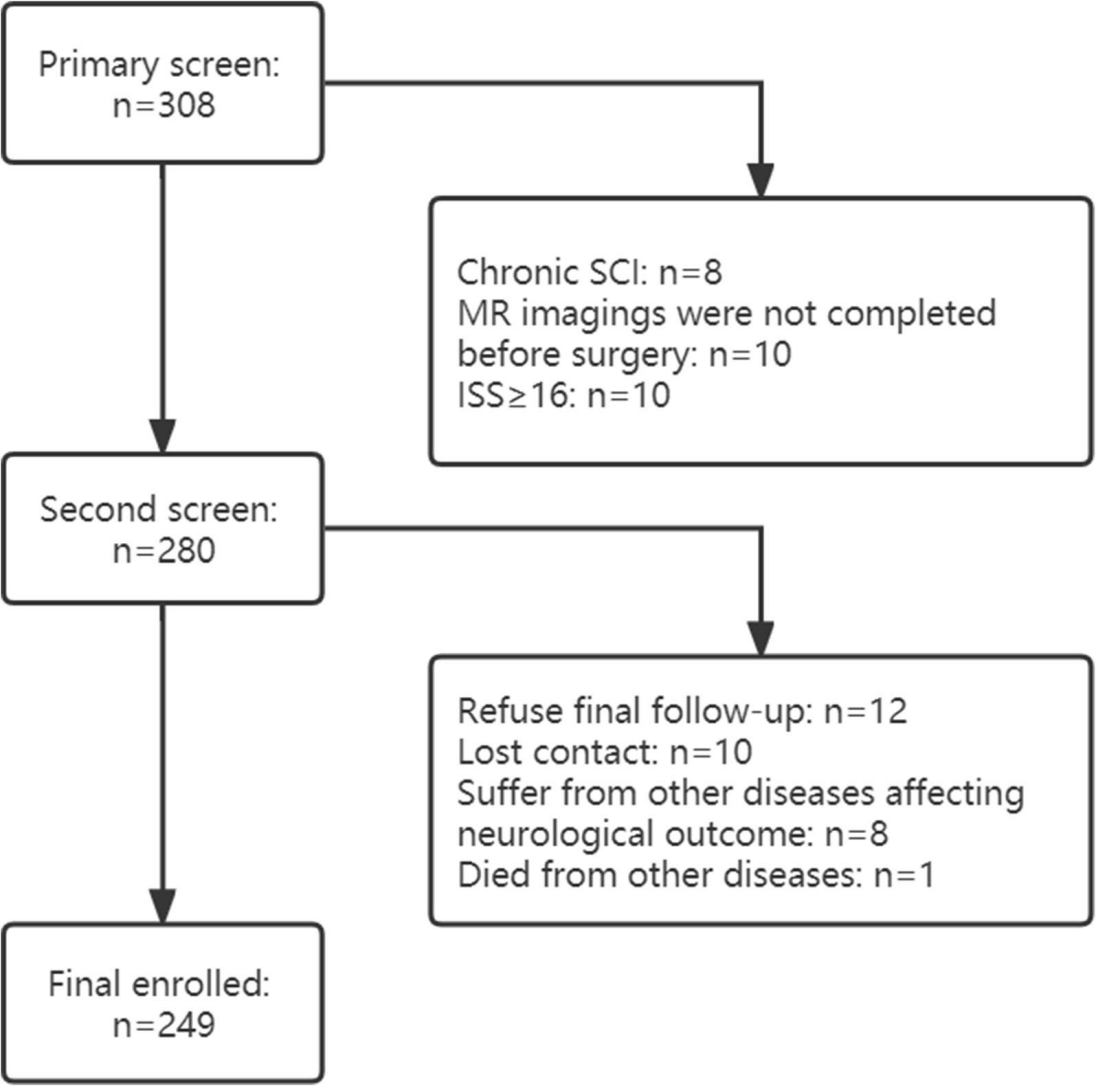
Patients flow

A total of 224 subjects were screened for enrolment of whom 249 satisfied study inclusion and exclusion criteria (Fig. 1). 48 patients were included in the validation sample set. The characteristics of the prospective population and the validation sample set are summarized in Table 2. The average age of the patients was 50.45 years, most of them were male, and the main injury was to the cervical segment. D-grade patients accounted for nearly half of the number, and there were 59 A-grade patients. Approximately one-third of patients received surgical decompression within 24 h.

**Table 2:** Population characteristics

	Model building set	The validation sample set
Age	50.45 ± 14.91	47.1 ± 11.39
Sex	Male = 182; Female = 67	Male = 35; Female = 13
Level of injury	Cervical = 193; Thoracic = 56	Cervical = 38; Thoracic = 10
AIS at admission	A = 59; B = 13; C = 50; D = 127	A = 14; B = 6; C = 16; D = 12
Baseline AMS	63.51 ± 28.6	55.58 ± 27.57
BASIC score	Score 1 = 134; Score 2 = 60; Score 3 = 29; Score 4 = 26	Score 1 = 13; Score 2 = 20; Score 3 = ; Score 4 = 8
Time to operation	Early surgery = 89; Delayed surgery = 160	Early surgery = 22; Delayed surgery = 26
SCIM score	80.9 ± 22.76	76.28 ± 26.66

### Modeling and validation

**Fig. 2:**
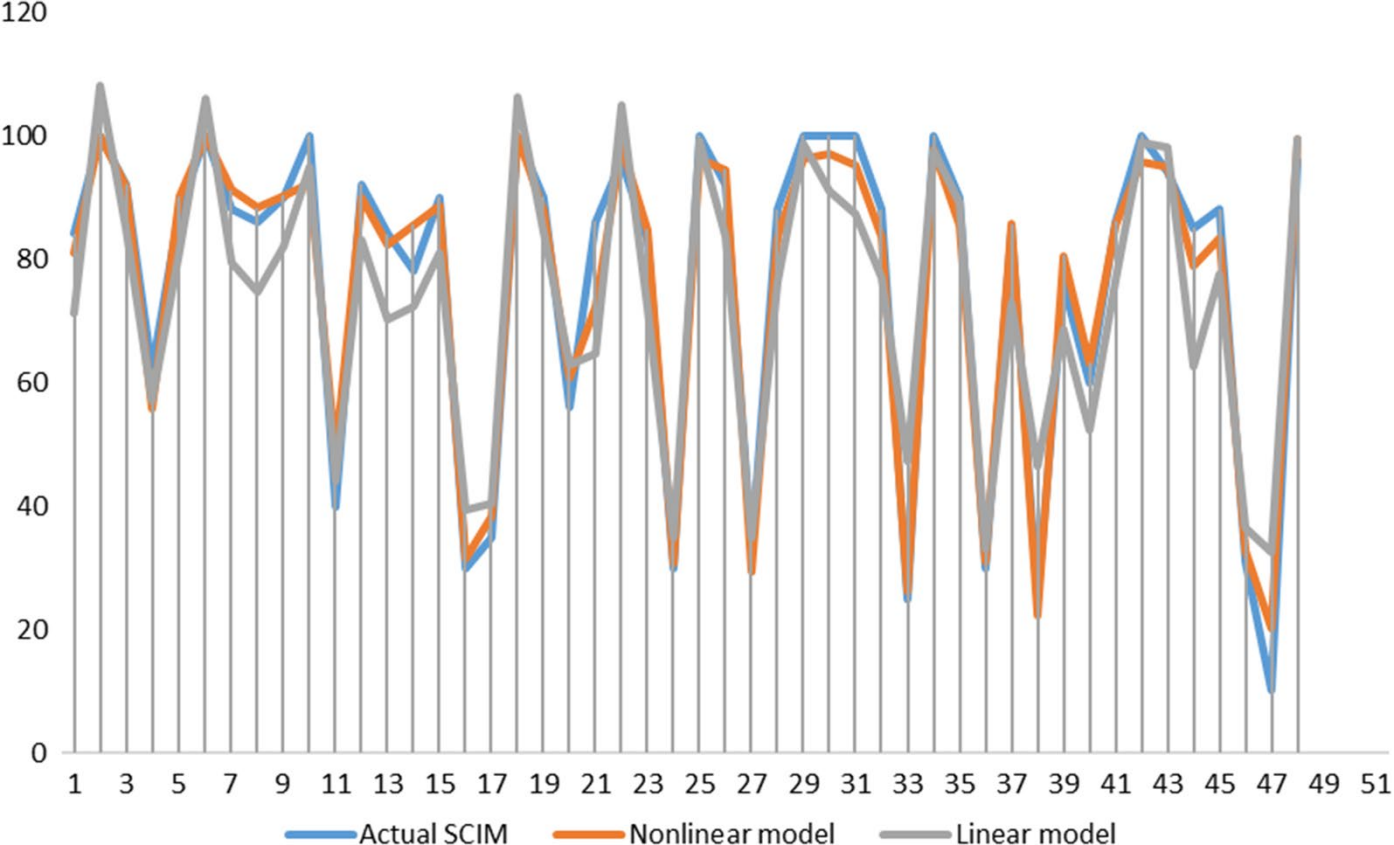
Validation of predictive model. Comparison between actual value, nonlinear model and linear model predicted value

XGBoost was used to build the nonlinear regression predictive model, and the coding data are shown in Additional file 1. The first 10 CARTs are shown in Additional file 2. The linear regression predictive model equation is SCIM III = 79.42—0.14*age + 6.3*surgical time (Delayed surgery = 1; Early surgery = 2)—1.17*injury level (Thoracic = 1; Cervical = 2) + 0.23*AMS + 3.45*AIS at admission (A = 1; B = 2; C = 3; D = 4; E = 5) -12.6*BASIC score (score 1 = 1; score 2 = 2; score 3 = 3; score 4 = 4). The validation sample set was used to verify the nonlinear regression prediction model (*t* = 0.86, *P* = 0.394; Mean ± SD: 3.31 ± 2.8) and linear regression prediction model (*t* = 1.83, *P* = 0.074; Mean ± SD: 8.61 ± 5.69). The MSE MAE and MAPE of the nonlinear predictive model were 18.59 3.01 and 3.22, while the MSE MAE and MAPE of the linear regression predictive model were 105.88 8.61 and 8.7. The paired sample T test indicated that the difference between the two groups (the absolute value of the difference between the predicted value of the nonlinear regression model and the real value; the absolute value of the difference between the predicted value of the linear regression model and the real value) was 5.31 (95% CI: 3.68–6.93), with a significant difference (*t* = 6.57, *P* < 0.001), suggesting that the prediction accuracy of the nonlinear regression model is better than that of the traditional linear regression model (Fig. 2).

### Other findings

**Fig. 3:**
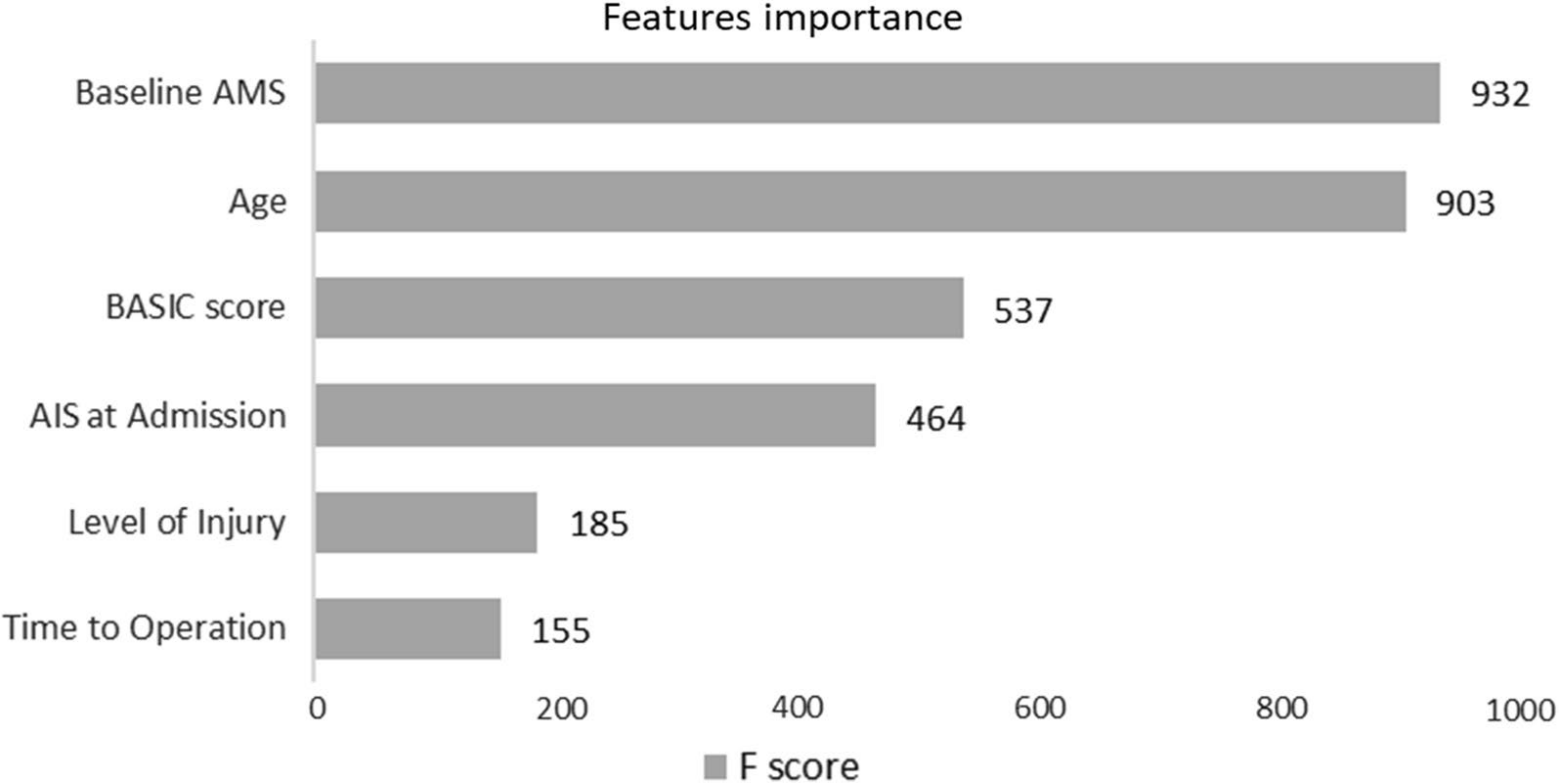
Rank of features importance

**Fig. 4:**
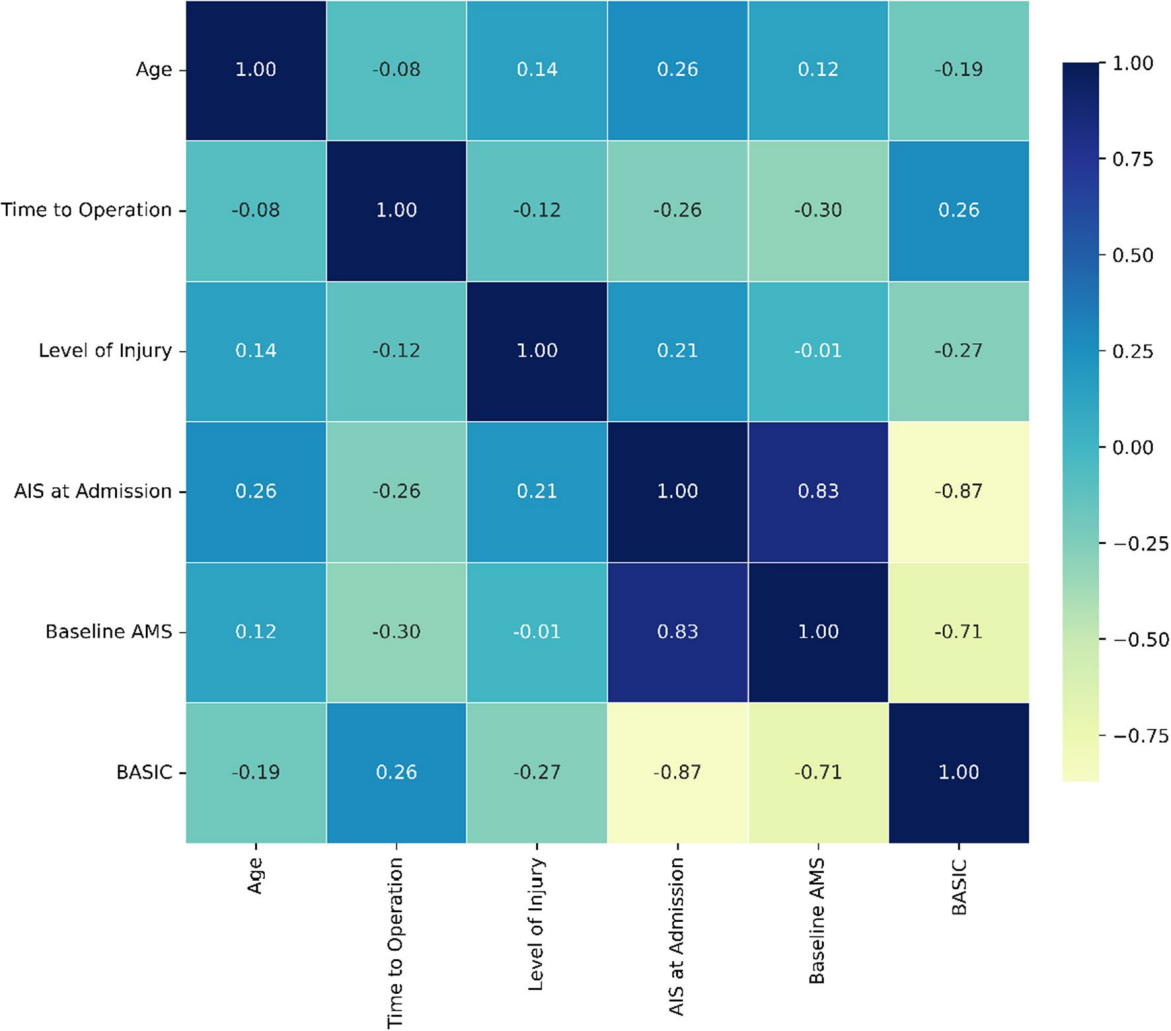
Correlation of the 6 predictors

Moreover, we ranked the importance of features in constructing predictive models (Fig. 3). We found that AMS and age played the most important roles in constructing predictive models. The correlation between the 6 predictors is shown in Fig. 4. There is an obvious correlation between AIS, AMS and BASIC score.

## Discussion

We prospectively enrolled 249 patients with acute SCI from 5 primary orthopedic centers. Based on 6 predictors with three aspects (age, AIS at admission, baseline AMS, level of injury, BASIC score and surgical timing), we successfully constructed a nonlinear regression prediction model through XGBoost and verified the credibility.

Acute SCI has always been the focus of clinicians due to its high incidence and high disability rate. Early prediction of the functional prognosis of patients is conducive to guiding follow-up treatment and giving the patients and their families a realistic idea of long-term expectations. Wilson [6] and Kaminski et al [7]. constructed linear regression models based on similar clinical features and MR images in 2012 and 2017, respectively. However, much progress has been made in understanding the injury mechanism, clinical features, MR images, and treatment options. First described by Talbott et al. [19], the BASIC score have proved the value in assessing the SCI severity and predicting SCI prognosis [20, 21]. Haefeli [21] and Mabray [20] also found the BASCI score is superior to the other MR imaging measures. At the same time, surgical decompression has been considered the most effective treatment for acute SCI. Many clinical trials have shown that early surgery [8–11] improve the prognosis of patients, and these measures have been included in the guidelines [26]. Therefore, in order to obtain a better prediction of the neurological prognosis of patients with acute SCI, we believe it is necessary to incorporate the above predictors as a supplement to the previous prediction models.

As the most advanced technology in machine learning at present [13], XGBoost has been widely used in various fields, such as industry, commerce and environmental protection, to construct nonlinear regression models. It was also used to build in-hospital mortality prediction models for patients with acute coronary syndrome and performed better than traditional linear regression models [27–29]. Therefore, we used XGBoost technology to incorporate representative data into the analysis to construct a nonlinear regression prediction model for the functional outcome of patients with acute SCI.

In the process of constructing the predictive model, we counted the importance of each feature’s value in predicting the patient’s functional outcome. AMS has been found to play the most important role in predicting the functional outcome of patients, while AIS is relatively less important. In previous studies, both AMS and AIS were considered to be related to the improvement of patients’ neurological function [6, 7]. We believe that there are broad differences in AMS in the same AIS grade. AIS is a hierarchical grading index, while AMS refers to the accumulation of key muscle group strength grading, which is a continuous variable [23]. The SCIM score is composed of 19 items with three main domains: self-care, respiration and sphincter management and mobility [24]. The realization of each function is closely related to the strength of key muscle groups, so AMS plays a more critical role in predicting functional outcome. This can also verify that AIS alone is less effective in judging the functional outcome of patients [30, 31].

We found that age also played an important role in predicting functional outcome. Age has always been wildly considered to be significantly related to the improvement of patients’ neurological function [32–36]. Through our study, we determined that age is the second most important influencing factor of neurological prognosis in acute SCI patients, after AMS. However, the surgical timing ranks last in importance, which suggests that the surgical timing may have a relatively low impact on the functional prognosis of patients with SCI. This shows that the functional recovery after SCI is more closely related to the severity of the injury and the age of the patient, while the timing of surgery can only have a small impact. This research conclusion does not mean that early surgery is not beneficial to the improvement of patients' neurological function and does not conflict with previous clinical studies. The reason is that the improvement of neurological function in acute SCI patients is often defined as the change in the postoperative AIS grade compared with the AIS at admission. In our prediction model, the functional prognostic indicator was the SCIM score at 1 year after surgery, rather than the change. In fact, in our study results, we found that the SCIM score at 1 year after surgery of patients in the early surgery group was significantly lower than that in the delayed surgery group. We believe that this may be because patients who underwent early surgery tended to have more severe injuries and lower baseline AMS.

Through correlation analysis, we found that there was a significant correlation between AIS grade, BASIC score and AMS. When patients have combined injuries, such as combined fractures, pain, and brain injuries, it is very important to assess the severity of the patient's SCI through MR. Multiple clinical studies have proven that the BASIC score has a significant correlation with the severity of SCI and predicting functional improvement [20, 21]. Our research also further supports the view that there was a significant correlation between AIS grade, BASIC score and AMS. This confirms the value of BASCI score in assessing the severity of acute SCI.

### Limitations

(1) The sample size data were insufficient. This is one of the largest prospective studies about constructing a model for predicting the functional outcome of acute SCI, but for machine learning, the sample size should be as large as possible. (2) The validation set data were collected retrospectively, while the model we built was based on a prospective study. (3) The constructed prediction model can only be stored in the form of an algorithm, which limits its promotion and extensive verification. (4) A small proportion of the clinical data were not collected within 72 h, such as MR measures which would be affected by the time. (5) The SCI segment is simply divided into cervical and thoracic vertebrae, which is relatively coarse. Some studies have reported that upper thoracic SCI has worse neurologic prognostic potential than thoracolumbar SCI.

## Conclusions

We verified the feasibility of using XGBoost to construct a nonlinear regression prediction model for the functional outcome of patients with acute SCI, and proved that the predictive performance of the nonlinear model is better than the traditional linear regression prediction model. Age and baseline AMS play the most important role in predicting the functional outcome. We also found a significant correlation between AIS at admission, baseline AMS and BASIC score. However, due to the limitations mentioned above, it is necessary to conduct more extensive and in-depth research.

## Supplementary Information

**Additional file 1.** Coding data.

**Additional file 2.** The first 10 Classification and Regression Trees (CARTs).

## Data Availability

The datasets generated and analyzed during the current study are not publicly available due to the data also forms part of an ongoing study but are available from the corresponding author on reasonable request.

## Code availability

Concluded in the supplementary materials.
