# Supplementary material for: A functional outcome prediction model of acute traumatic spinal cord injury based on extreme gradient boost
Source: J Orthop Surg Res. 2022 Oct 12;17:451. doi: 10.1186/s13018-022-03343-7 (PMC9559032; doi:10.1186/s13018-022-03343-7)
Supplement: Supplementary file 1 — Additional file 1. Coding data. [file 13018_2022_3343_MOESM1_ESM.docx]

import pandas as pd

import numpy as np

import xgboost

def dataprocess(dataset):

categorical_features = [ 'Time to Operation', 'Level of Injury', 'AIS at Admission', 'BASIC']

every_column_non_categorical= [col for col in dataset.columns if col not in categorical_features and col not in ['Patient ID', 'SCMI 1year'] ]

numeric_feats = train[every_column_non_categorical].dtypes[dataset.dtypes != "object"].index

dataset[numeric_feats] = np.log1p(dataset[numeric_feats])

dataset = pd.get_dummies(dataset,columns =categorical_features)

return dataset

train = pd.read_csv('./train.csv', sep='\t', header=0)

test = pd.read_csv('./test.csv', sep='\t', header=0)

train_test = pd.concat([train, test], keys=['train', 'test'])

train_test = dataprocess(train_test)

train, test = train_test.loc['train'], train_test.loc['test']

model = xgboost.XGBRegressor(colsample_bytree=0.4,

gamma=0,

learning_rate=0.07,

max_depth=3,

min_child_weight=1.5,

n_estimators=10000,

reg_alpha=0.75,

reg_lambda=0.45,

subsample=0.6,

seed=42)

every_column_except_y= [col for col in train.columns if col not in ['Patient ID', 'SCMI 1year']]

model.fit(train[every_column_except_y],train['SCMI 1year']/100.0)

model.save_model('xgb.model')

from xgboost import plot_tree

import os

import matplotlib.pyplot as plt

plt.rcParams['savefig.dpi'] = 600

plt.rcParams['figure.dpi'] = 1080

plt.figure(figsize=(15, 15))

# print(model.num_parallel_tree)

for i in range(10):

plot_tree(model, num_trees=i, rankdir='LR')

plt.savefig(f'tree{i}.svg', format='svg')

plt.show()

# plot_tree(model, num_trees=1, rankdir='LR')

# plt.savefig('tree1.svg', format='svg')

# plt.show()

test['Prediction'] = np.clip(model.predict(test[every_column_except_y]) * 100, 0, 100)

pd.DataFrame({'Patient ID': test['Patient ID'], 'SCMI 1year':test['SCMI 1year'], 'Prediction': test['Prediction']}).to_csv('result.csv',index=False)

import xgboost as xgb

from xgboost import plot_importance

import matplotlib.pyplot as plt

plot_importance(model)

plt.show()

import matplotlib.pyplot as plt

import seaborn as sns

def correlation_heatmap(train):

correlations = train.corr()

fig, ax = plt.subplots(figsize=(10,10))

sns.heatmap(correlations, vmax=1.0, center=0, fmt='.2f', cmap="YlGnBu",

square=True, linewidths=.5, annot=True, cbar_kws={"shrink": .70}

)

plt.show()

trainX = pd.read_csv('./train.csv', sep='\t', header=0)

correlation_heatmap(trainX.loc[:, [col for col in trainX.columns if col not in ['Patient ID', 'SCMI 1year']]])

#input type: data = {'Age': 20, 'Time to operation': 1, 'Level of injury': 1, 'AIS at admission': 1, 'Baseline AMS': 18, 'BASIC': 3}

def predict(data):

model = xgboost.XGBRegressor()

model.load_model('xgb.model')

input = []

input.append(np.log1p(data['Age']))

if data['Time to operation'] == 1:

input.extend([1, 0])

else:

input.extend([0, 1])

if data['Level of injury'] == 1:

input.extend([1, 0])

else:

input.extend([0, 1])

if data['AIS at Admission'] == 1:

input.extend([1, 0, 0, 0])

elif data['AIS at Admission'] == 2:

input.extend([0, 1, 0, 0])

elif data['AIS at Admission'] == 3:

input.extend([0, 0, 1, 0])

elif data['AIS at Admission'] == 4:

input.extend([0, 0, 0, 1])

input.append(np.log1p(data['Baseline AMS']))

if data['BASIC'] == 1:

input.extend([1, 0, 0, 0])

elif data['BASIC'] == 2:

input.extend([0, 1, 0, 0])

elif data['BASIC'] == 3:

input.extend([0, 0, 1, 0])

elif data['BASIC'] == 4:

input.extend([0, 0, 0, 1])

input = np.array([input])

ypred = model.predict(input)

return ypred[0] * 100

data = {'Age': 20, 'Time to operation': 1, 'Level of injury': 1, 'AIS at Admission': 1, 'Baseline AMS': 18, 'BASIC': 3}

y = predict(data)

y
