# Supplementary figures and images for: A functional outcome prediction model of acute traumatic spinal cord injury based on extreme gradient boost
Source: J Orthop Surg Res. 2022 Oct 12;17:451. doi: 10.1186/s13018-022-03343-7 (PMC9559032; doi:10.1186/s13018-022-03343-7)

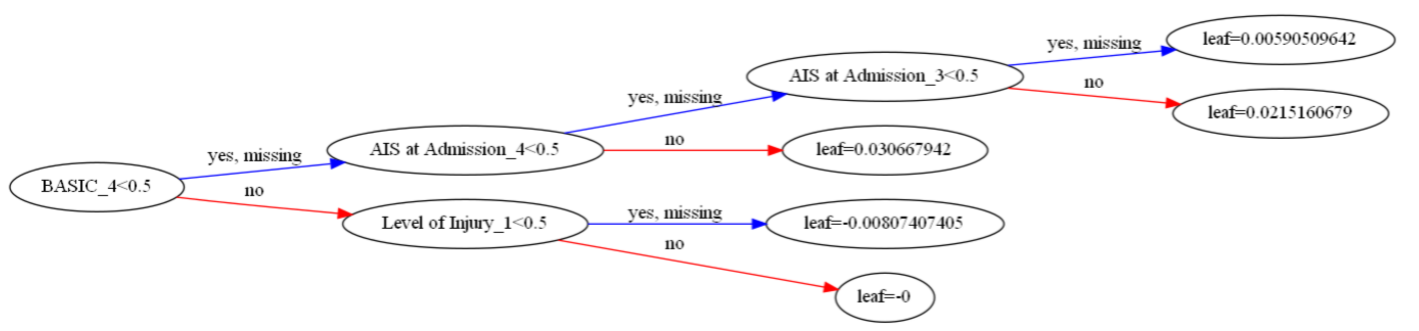


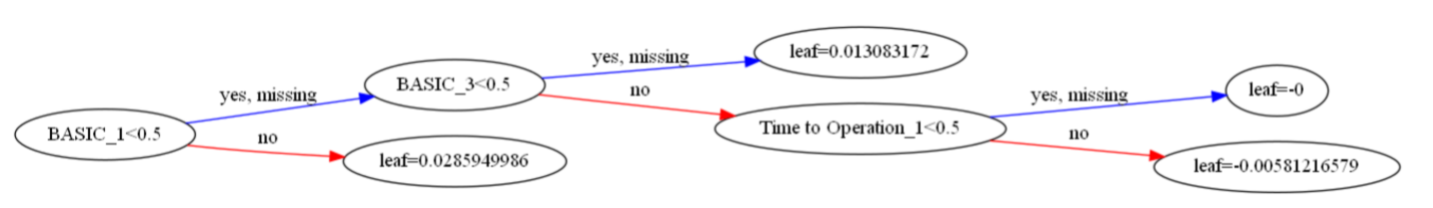


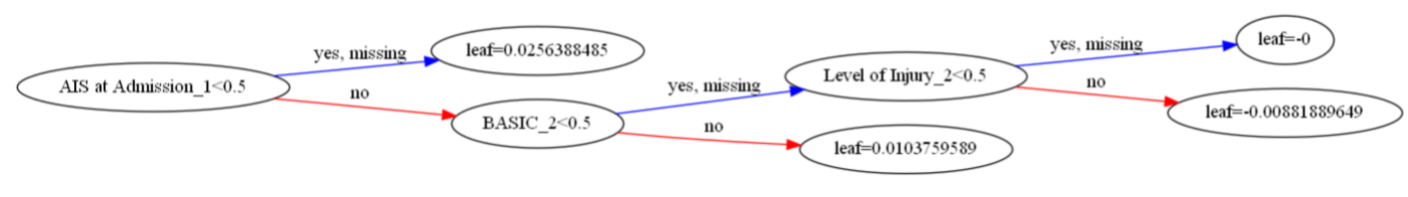


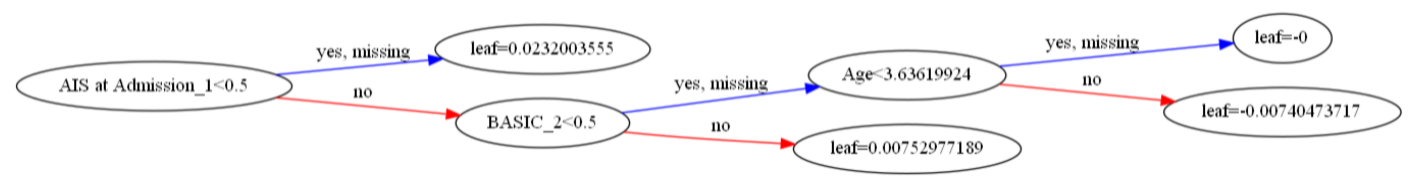


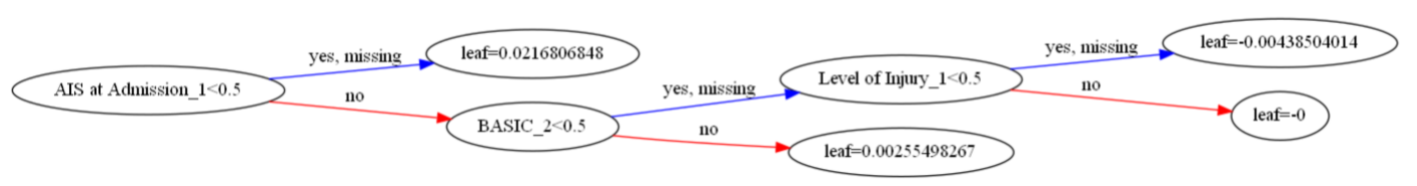


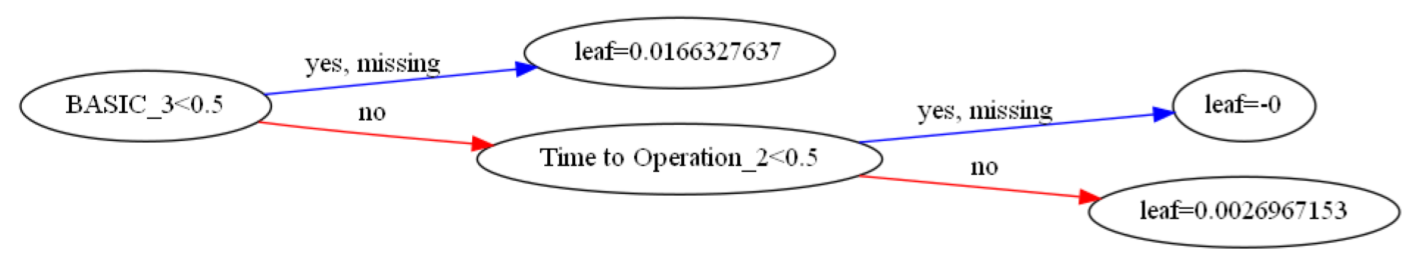


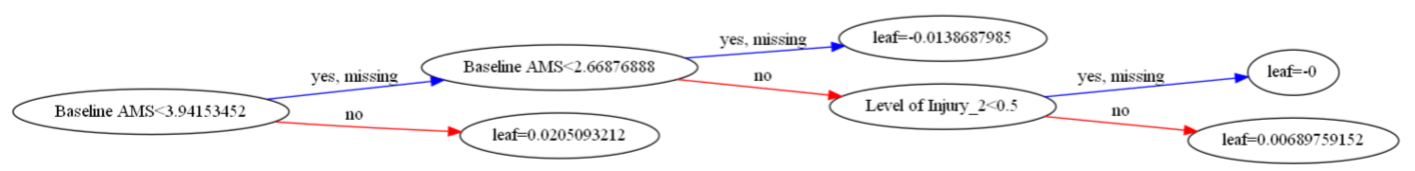


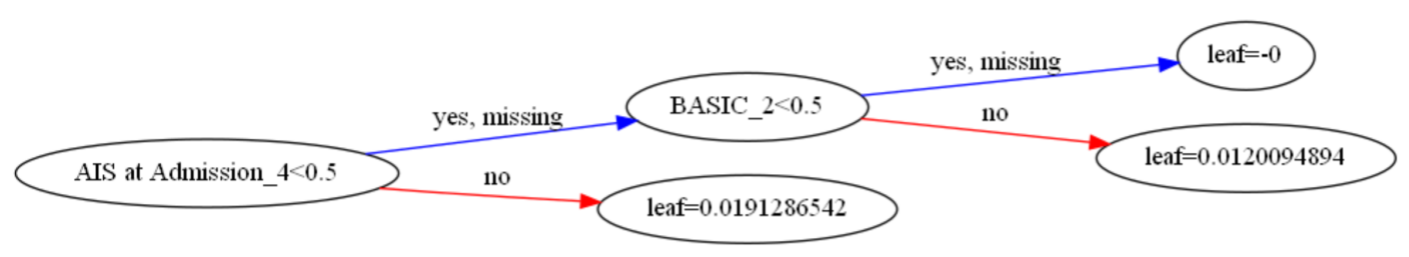


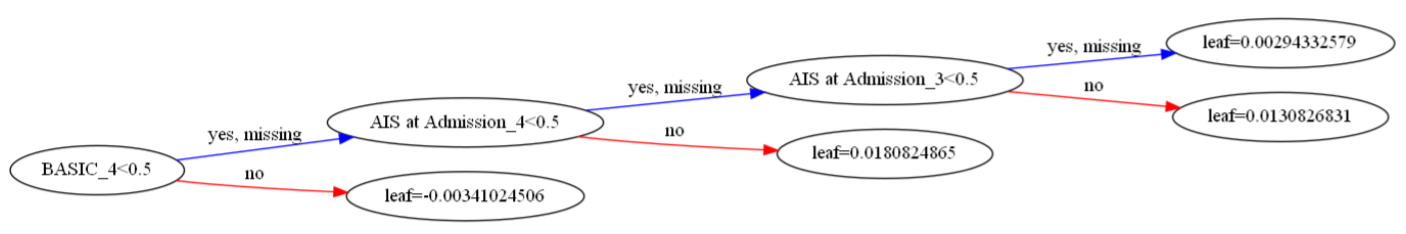


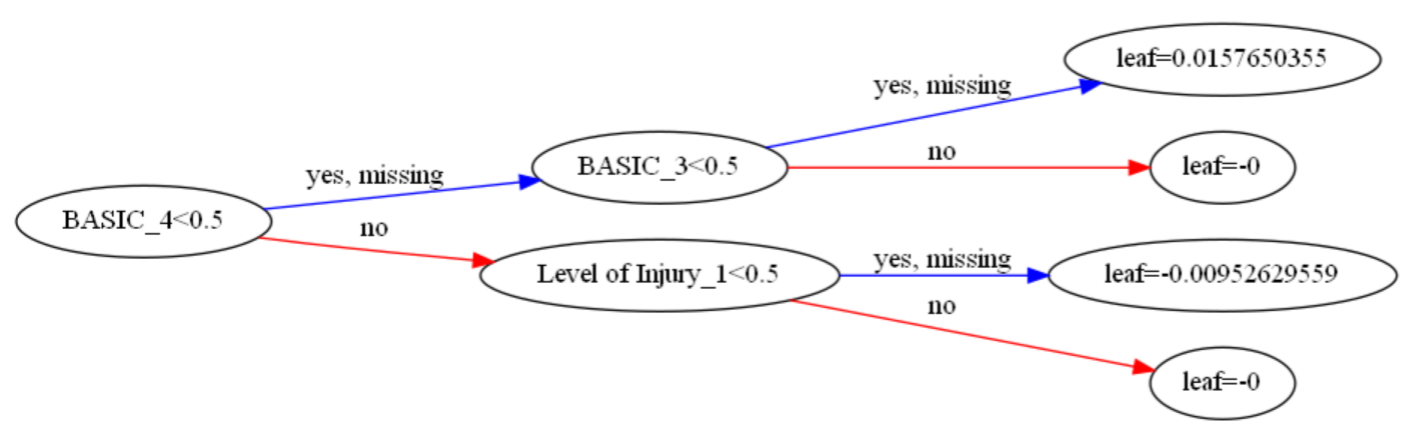

Supplement: Supplementary file 2 — Additional file 2. The first 10 Classification and Regression Trees (CARTs). [file 13018_2022_3343_MOESM2_ESM.docx]
